# Bone Indices in Thyroidectomized Patients on Long-Term Substitution Therapy with Levothyroxine Assessed by DXA and HR-pQCT

**DOI:** 10.1155/2015/796871

**Published:** 2015-07-13

**Authors:** Emil Moser, Tanja Sikjaer, Leif Mosekilde, Lars Rejnmark

**Affiliations:** Department of Endocrinology and Internal Medicine, THG, Aarhus University Hospital, 8000 Aarhus C, Denmark

## Abstract

*Background*. Studies on bone effects of long-term substitution therapy with levothyroxine (LT_4_) have shown discrepant results. Previous studies have, however, not evaluated volumetric bone mineral densities (vBMD), bone structure, and strength using high resolution peripheral quantitative computed tomography (HR-pQCT) and finite element analysis (FEA). Using a cross-sectional design, we aimed to determine whether BMD, structure, and strength are affected in hypothyroid patients on LT_4_ substitution therapy.* Methods*. We compared 49 patients with well-substituted hypothyroidism with 49 age- and gender-matched population based controls. Areal BMD was assessed by DXA, vBMD and bone geometry by HR-pQCT, and bone strength by FEA.* Results*. Patients had been thyroidectomized due to thyroid cancer (10%) and nontoxic (33%) or toxic goiter (57%). 82% were women. TSH levels did not differ between groups, but patients had significantly higher levels of T4 (*p* < 0.001) and lower levels of T3 (*p* < 0.01). Compared to controls, patients had higher levels of magnesium (*p* < 0.05), whereas ionized calcium and PTH were lower (*p* < 0.05). Bone scans did not reveal any differences in BMD, bone geometry, or strength.* Conclusion*. If patients with hypothyroidism are well-substituted with LT_4_, the disease does not affect bone indices to any major degree.

## 1. Introduction

Thyroid status is known to affect bone metabolism. Bone turnover is decreased in hypothyroidism, whereas hyperthyroidism increases bone turnover, bone loss, and risk of fracture [1–6]. High levels of thyroid hormones increase the activation of new remodeling cycles in bone and lead to a shortening of the cycle [7, 8].* In vitro* studies have shown an important role of T3 as it stimulates osteoblastic differentiation thereby promoting matrix synthesis and mineralization [9]. However, T4 can be converted to T3 locally in the bone tissue by deiodinase type 2 [10], but little is known of the relative importance* in vivo* of T4 and T3 levels in blood.

Patients with hypothyroidism normally receive replacement therapy with levothyroxine (LT_4_). Dose of LT_4_ is titrated according to plasma levels of thyroid-stimulating hormone (TSH). In patients with hypothyroidism due to nonmalignant causes, the dose of LT_4_ is titrated in such a manner that TSH levels are within the lower range of the reference interval (replacement therapy). In patients with a history of thyroid cancer, a relatively high dose of LT_4_ is often given initially aiming at slightly suppressing TSH levels (TSH suppression therapy). However, after a certain number of years without recurrence, treatment strategy is often changed to replacement therapy [11]. Despite normal TSH levels, replacement with LT_4_ is often associated with relatively high T4 levels and an increased T4/T3 ratio. Although numerous studies have been performed, it has not yet been fully clarified whether risk of fracture [3, 12–14], bone density [15–17], and bone architecture [15] is affected in patients on long-term LT_4_ replacement therapy.

High-resolution peripheral quantitative computed tomography (HR-pQCT) is a new technique which allows for* in vivo* assessment of vBMD as well as bone geometry and microarchitecture at the distal radius and tibia. Using this technique along with conventional DXA scans and biochemical bone markers, we aimed to determine whether long-term replacement therapy with LT_4_ affects bone.

## 2. Subject and Methods

Using a cross-sectional study design, we compared 49 patients on long-term treatment with LT_4_ with 49 age- and gender-matched controls. We identified 219 patients who had undergone thyroid surgery (total/partial thyroidectomy) at our Hospital from 1971 to 2009. The mean duration from surgery to inclusion was 11 years. We considered men and women between 18 and 80 years eligible for inclusion. Patients were only included if they had received continuously LT_4_ treatment for more than 2 years. To ensure that patients were well-treated at the time of inclusion, we evaluated earlier blood samples. Patients were considered well-treated, if they have had at least one measurement of plasma TSH within the reference range and none above or below the range during the last 6 months prior to inclusion. To identify controls we used the Danish Civil Registration System which generated a list providing us with name and address of 100 persons of mixed gender for each birth year between 1932 and 1982, giving a total of 5000 individuals. These individuals were selected randomly from the population living in the vicinity of Aarhus city, Denmark. When a hypothyroid patient was included, 5–10 gender- and age- (±2 years) matched controls were invited by letter to participate in a random basis. Controls meet the same inclusion and exclusion criteria as patients. We excluded participants with hypo- or hyperparathyroidism, severely impaired renal function (estimated GFR <30 mL/min), and a history of malignant disease within 2 years and participants hospitalized due to chronic alcohol or drug abuse. Other exclusion criteria comprised sarcoidosis, pregnancy, and untreated malabsorption. Finally we excluded participants treated with lithium, amiodarone, systemic corticosteroids, and activated vitamin D analogues, as well as participants using a daily supplement of more than 1200 mg calcium or more than 50 mcg of vitamin D (D_2_ or D_3_). Moreover, controls with a prior history of thyroid disease or a plasma TSH outside the reference range were excluded. Participants provided written informed consent. The study was performed in accordance with the Declaration of Helsinki II and approved by The Central Denmark Region Committees on Health Research Ethics (M-20110260), the Danish Data Protection Agency (2007-58-0010), and the Central Denmark Region (1-10-72-76-12).

### 2.1. Biochemistry

Fasting blood samples were drawn in the morning. To avoid seasonal variation in 25-hydroxyvitamin D (25OHD) levels, samples from matched pairs of patients and controls were taken within a 2-week time-span. We measured plasma levels of albumin, calcium, phosphate, T4-uptake, magnesium, creatinine, and creatinine-kinase by standard laboratory methods. To reduce analytical variation, plasma levels of parathyroid hormone (PlTH), 25OHD, bone specific alkaline phosphatase (BSAP), procollagen I, N-terminal propeptide (PINP), osteocalcin, and C-terminal telopeptide of type I collagen (*β*-CrossLaps, CTx) were analyzed in a single batch. Serum samples were stored at −80°C until analyzed.

TSH, T3, and T4 were measured using electrochemiluminescence immunoassay (ECLIA) on an automated instrument (Cobas e 601, Roche Diagnostics, GmbH, Mannheim, Germany). For TSH, the lower detection limit is 0.005 × 10^3^ IU/L with a total imprecision (CV,%) of 8.6%, 2.1%, and 1.8% at serum values of 0.034 × 10^3^ IU/L, 0.91 × 10^3^ IU/L, and 3.96 × 10^3^ IU/L, respectively. For T3, the lower detection limit is 0.300 nmol/L with a CV% of 3.6%, 4.2%, and 5.3% at serum levels of 1.22 nmol/L, 2.87 nmol/L, and 5.09 nmol/L, respectively. The lower detection limit for T4 is 5.40 nmol/L with a CV% of 1.8%, 1.3%, and 1.7% at 63.1 nmol/L, 84.3 nmol/L, and 243 nmol/L, respectively.

Plasma intact PTH levels were measured using a 2nd generation (ECLIA) on an automated instrument (Cobas 6000, Roche Diagnostics, GmbH, Mannheim, Germany). The lower limit of detection was 0.127 pmol/L, with a CV% of 3.3% and 2.7% at serum values of 3.7 pmol/L and 26.6 pmol/L, respectively.

We analyzed PINP, osteocalcin, and CTx using enzyme-linked immunosorbent assay (ELISA) on an automated instrument (Cobas 6000, Roche). For PINP, the measuring range was 12.5–2400 *μ*g/L with a CV% of 1.64% at 65.8 *μ*g/L, 2.29% at 371.2 *μ*g/L, and 2.11% at 748.5 *μ*g/L. For osteocalcin, the lowest detectable value was 0.5 *μ*g/L, with a CV% of 0.97% and 1.06% at 25.3 *μ*g/L and 84.1 *μ*g/L, respectively. The lowest measurable level of CTx was 0.03 *μ*g/L. The CV% was 2.97% at 0.32 *μ*g/L and 1.51% at 2.77 *μ*g/L.

### 2.2. Osteodensitometry

DXA was performed using a Hologic QDR Discovery scanner (Hologic Inc., Waltham MA, USA). Areal bone mineral density (aBMD, gram/cm^2^) was measured at the spine (L1–L4), the right hip, the right forearm, and the whole body. We also evaluated body composition (lean- and fat-tissue mass). The CV% was 1.5% at the lumbar spine, 2.1% at the femoral neck, and 1.9% at the distal radius [18].

### 2.3. Bone Structure and Geometry

Volumetric BMD (vBMD, mg of hydroxyapatite/cm^3^) and microarchitecture were measured at the right distal radius and right distal tibia using a HR-pQCT scanner (XtremeCT, SCANCO Medical AG, Brüttisellen, Switzerland). We applied the standard protocol for* in vivo* measurements supplied by the manufacturer. A 3D segment was constructed from 110 parallel CT slices with an isotropic voxel size of 82 *μ*m. Scans were performed 9.5 mm and 22.5 mm from the distal endplate at radius and tibia, respectively, and extending proximal, giving a bone segment with a total length of 9.02 mm. Standard bone evaluation was performed using manufacturer's default software. The outcome variables used were vBMD for the entire bone segment (*D*_tot_), trabecular (*D*_trab_), meta trabecular (*D*_meta_), inner trabecular (*D*_inn_), and compact bone (*D*_comp_) as well as cortical thickness (Ct.Th, mm), mean total, mean cortical, and mean trabecular area (mm^2^). Further outcomes are trabecular bone volume/tissue volume fraction (BV/TV), number of trabeculae (Tb.N, 1/mm), trabecular thickness (Tb.Th, mm), and separation (Tb.Sp, mm). Also, standard deviation of 1/Tb.N (Tb.1/N.SD, mm) was used as a measure for inhomogeneity of network. Each scan was visually inspected and image quality was rated on a scale from 1 to 5 according to guidelines provided by Scanco Medical. Scans of poor quality were discarded, and a new scan was performed up to a total maximum of 3 scans to limit radiation dose.

### 2.4. Finite Element Analysis

Finite element analysis (FEA) was performed using a FEA solver v1.15 provided by Scanco Medical. In brief, FE models were created from the HR-pQCT scans mentioned earlier, by converting the measured voxels into isometric brick elements, resulting in FE models with approximately 2 and 5 million elements for radius and tibia, respectively. The materials were given linear elastic properties with Young's Modulus of 10 Gpa and Poisson's ratio of 0.3 for both cortical and trabecular bone. In accordance with the Pistoia criteria, it is assumed that bone failure occurs when more than 2% of the elements are strained beyond a critical level of 7000 *μ*strain [19]. Outcomes from this evaluation are failure load [N] and stiffness [N/mm].

## 3. Statistics

According to our sample size calculations 50 subjects would be needed in each study group to be able to detect a 2% difference between groups in aBMD of the lumbar spine at the 5% significance level, with a statistical power of 80%. We assessed differences between groups using nonparametric test for two or more independent groups in terms of either Mann-Whitney test or Kruskal-Wallis test. Bivariate correlations were assessed by Spearman's rho and multiple regression analyses were used to adjust for potential confounders. We performed all analyses using the IBM Statistical Package for Social Sciences (SPSS 20.0) for Windows.

## 4. Results

Table 1 shows characteristics of included subjects. Among patients, 57% had a history of toxic goiter, including Grave's disease and 33% had a history of nontoxic goiter, while 10% had been thyroidectomized because of thyroid cancer (none were on TSH suppression therapy at the time of study). Patients had been on treatment with LT_4_ for a median of 11 (range of 3–41) years. Patients and controls were well matched with no difference in age and gender (82% women). Significantly more patients than controls used supplements of calcium, vitamin D, and magnesium (Table 1).

### 4.1. Biochemistry

TSH levels did not differ between patients and controls, but patients had significantly higher levels of T4 and lower levels of T3, resulting in a significantly higher T4/T3 ratio (Table 2).  Table 3 shows results stratified by etiology of thyroid disease. TSH, T3, T4, and T4/T3 ratio did not differ between patient groups. However, T3 was significantly lower compared to controls in the goiter groups, whereas T4 levels and T4/T3 ratio were significantly higher in all patient groups. After adjustment for age, gender, and BMI, daily dose of LT_4_ did not correlate with plasma levels of TSH, T3, or T4 (data is not shown), although a positive borderline significant correlation was found between daily dose of LT_4_ and T4/T3 ratio (*p* = 0.06).

Compared with controls, patients had significantly higher plasma levels of magnesium (*p* < 0.05), whereas levels of ionized calcium and PTH were significantly lower (Table 2).

Stratification by etiology (Table 3) showed significantly higher levels of 25OHD in the thyroid cancer group compared with both control and nontoxic, group and the toxic group. This remained significant after adjustment for use of vitamin D supplements (*p* < 0.05). Levels of 25OHD correlated with vitamin D supplementation (*p* < 0.05). Daily dose of LT_4_ did not correlate with plasma levels of ionized calcium, and this was not changed by adjustment for age, gender, and BMI.

Biochemical markers of bone turnover did not differ significantly between groups (Table 2), and levels did not correlate to daily dose of LT_4_ or levels of TSH, T3, T4, or T4/T3 ratio. Nor did stratification by etiology or gender reveal any biochemical differences.

### 4.2. Body Composition

There were no differences in height, weight, and BMI between groups (Table 1). Moreover, body composition as assessed by DXA did not differ between groups (Table 4).

### 4.3. Bone Density, Structure, and Strength

DXA showed no differences between patients and controls in aBMD at the hip, spine, forearm, or whole body (Table 4). However, compared with their matched controls, patients with prior history of toxic goiter had a borderline significant lower aBMD in the whole body (1.102 [1.020–1.180] versus 1.160 [1.154–1.253], *p* = 0.06).

The HR-pQCT scans did not show differences in vBMD or bone structure between patients and controls (Table 5). After adjustment for age, gender, and BMI and stratification by etiology, patients with a history of thyroid cancer had a significantly thicker cortex in the radial bone than both previous nontoxic and toxic patients. Furthermore, they had a lower mean total area but a greater mean cortical area than the nontoxic group (Table 3). Total vBMD at the radius was higher in thyroid cancer group, than in the toxic goiter group. However, stratification by etiology of hypothyroidism did not reveal differences between any of the three groups and their matched controls (Table 3). Furthermore, stratification by gender did not change the results.

Failure load and bone stiffness did not differ between groups. Higher T3 correlated to a higher failure load at the radius (*p* = 0.005) and tibia (*p* = 0.01), but this association was no longer significant after adjustment for age, gender, and BMI.

None of the bone indices correlated with length of LT_4_ treatment.

## 5. Discussion

In a cross-sectional study, we aimed to assess whether bones are affected in patients with postoperative hypothyroidism, which are well-treated by LT_4_ replacement therapy. The reason for studying this is that although patients are considered to be well-treated if TSH levels are normal, replacement therapy does not fully restore the normal balance between T4 and T3. As thyroid hormones are known to affect bone metabolism, even subtle changes in the balance between T3 and T4 may be of importance. However, our study showed no deleterious effect of LT_4_ replacement treatment on measured indices.

In thyrotoxicosis or subclinical hyperthyroidism, bone turnover is increased as measured by bone turnover markers. Histomorphometric studies of iliac crest bone biopsies have shown an increased activation frequency with a shortened duration of each remodeling cycle. Bone resorption is increased to a greater extent than bone formation resulting in decreased BMD [7, 8]. Moreover, thyroid hormone levels within the euthyroid range may also affect bone. Murphy et al. [20] reported an inverse association between aBMD levels of T4 and T3 in a group of euthyroid postmenopausal women. Similarly, van Der Deure et al. [21] reported an inverse association between aBMD and T4 (but not T3) levels in a group of elderly men and women, and Roef et al. [22] have recently shown a similar inverse association between aBMD and T3 levels in young healthy euthyroid men. The relative importance of T4 and T3 hormones on bone has not yet been entirely elucidated. Circulating levels of T3 may not fully reflect the effect of thyroid status on bone, as osteoblasts express the type 2 deiodinase enzyme and may locally convert T4 into T3 [10]. T3 is considered to be of predominantly importance to bone by binding to thyroid hormone receptor *α* (TR*α*) on osteoblastic cells thereby promoting osteoblast differentiation with an increased osteoid matrix synthesis and mineralization [9]. In addition, T3 increases bone resorption. Most likely its actions are indirect through its effects on osteoblasts, but it is unclear whether this occurs through an increased expression of RANKL [23, 24]. In general, thyroid hormones are considered to be exerting anabolic effects during growth and development, whereas net catabolic effects occur in adulthood [25].

Moreover, it is also unclear whether thyroid hormones affect cortical and trabecular bone differentially [26]. In most studies, BMD, at skeletal sites rich in cortical bone, has been shown to vary inversely with levels of thyroid hormones, whereas variable effects have been reported on skeletal sites rich in trabecular bone [20–22, 27]. Previously histomorphometric studies by Mosekilde and Melsen [28] on hyperthyroid patients showed no effect on the osteocytes but they found an increase in the osteoclastic resorption surfaces on both trabecular and cortical bone, but most dominantly in cortical bone. The remodelling space is increased in hyperthyroidism, which is followed by a significant loss of both cortical and trabecular bone. There was no effect on cortical width, but an increased cortical porosity, which was shown to decrease after 4 months of antithyroid treatment [29, 30].

Schneider et al. [15] compared vBMD in patients on long-term LT_4_ therapy with a group of matched controls. Patients with a history of thyroid cancer were on TSH suppression therapy, whereas patients on LT_4_ replacement therapy due to nontoxic goiter showed normal thyroid function except slightly increased T4 levels. Although vBMD at the distal radius did not differ at baseline, women on TSH suppression therapy experienced a significant decrease in vBMD at the distal radius during one-year follow-up. The decrease in total vBMD occurred despite a slightly increased trabecular vBMD indicating that the decrease was attributable to a decreased cortical vBMD. In order to improve our knowledge on effects of LT_4_ treatment on different bone compartments, we used a more advanced scanning technique with a higher resolution that allows for assessment of density in the individual bone compartments as well as providing information on bone architecture. Despite higher T4 levels and T4/T3 ratio in patients compared with controls, our study showed no alterations in measured indices, and neither was bone turnover affected as measured by biochemical bone markers. To the best of our knowledge, bone structure in patients with thyroid diseases has not previously been assessed by HR-pQCT scans. The resolution is much higher than standard pQCT, allowing a more detailed description of the bone microstructure. We thought that though aBMD may not be affected due to long-term LT_4_ treatment, it is possible that bone architecture and mechanical properties may be affected. Our results, however, did not indicate such alterations.

We found that patients with prior thyroid cancer had an increased cortical thickness and a larger mean cortical area in the radial bone. Furthermore, mean total bone area was smaller with a higher vBMD. The lower total area may suggest that the prior thyroid suppressive therapy may have enhanced periosteal resorption thereby reducing the size of the bone, while the increased cortical area and thickness suggest a relatively lower proportion of trabecular to cortical bone resulting in a higher vBMD. Our data do not allow for causal explanations on these changes in bone geometry although it may be speculated that long-term (iatrogenic) hyperthyroidism may result in an increased endosteal apposition following normalization of thyroid function.

Finite element analysis did not show any differences in bone stiffness or failure load between groups, which adds to our general findings that LT_4_ treatment does not impair bone structure. Nor did DXA scans reveal differences between patients and controls in aBMD. Apparently, whatever the effect on bone hyperthyroidism has prior to surgery, these changes are not detectable or have reversed to normal over time. Accordingly, the shown subtle changes in levels of thyroid hormones do not seem to be of any major importance to bone.

Our study showed no difference in body composition. This is in contrast to a recent study including 941 healthy male siblings in whom levels of thyroid hormones correlated positively with whole body fat mass and inversely with lean-tissue mass, although no associations between TSH and body composition were found [31]. Accordingly, it seems that small variation within the euthyroid range may affect body composition in healthy subjects. We cannot exclude that our sample size was too small to detect such small changes in fat- and lean-tissue mass.

### 5.1. Biochemistry

Patients had higher levels of plasma magnesium and a tendency towards higher levels of plasma 25OHD. This corresponds very well with intake of supplements. The patients are more prone to take calcium, vitamin D, and magnesium supplementation due to having a chronic disease and thereby close relations to the health care system.

Interestingly, our study showed that both plasma levels of PTH and ionized calcium were lower in patients than in controls. As all our studied patients had undergone total or partial thyroidectomy, we cannot exclude that removal or damage to one or more of the parathyroid glands during extensive surgery may have caused a relative hypoparathyroidism.

## 6. Strength and Limitations

Patients and controls were well matched by gender and age and patients and matched controls participated within 2 weeks of each other in order to avoid seasonal variation in blood levels of 25OHD. To avoid confounding, we excluded subjects with diseases, as well as subjects taking medications known to have an effect on either bone or the thyroid axis. Controls were recruited on a random basis. We did not acquire any health information up until final enrolment, making the population as representative as possible.

Limitations include potential selection bias. There is a possibility, due to the nature of voluntary participation, that controls are not representative to the entire Danish population. Some might have family members diagnosed with osteoporosis, which could motivate participation. If this was the case, a deleterious effect of LT_4_ on bone could be masked. Another possibility could be the “healthy worker effect.” However, for ethical reasons, we are not allowed to address those patients and controls who did not want to participate in the investigation. The patient group consists of different diseases, such as previous cancer, toxic goiter, and atoxic goiter and the question is whether they are comparable. Even though we did not find any statistical difference in BMD, bone turnover markers, or thyroid hormone status, we cannot rule out the possibility of undetectable differences between patients with Grave's disease having low TSH for a short time and patients with previous cancer and low TSH for a longer period due to suppressive therapy.

Our population was relatively small, comprising a total of 98 subjects. A larger population would have provided more power to the study, and thereby making it possible to detect minor differences between groups. However, if major differences were present in bone structure or density, our sample size was large enough to detect such differences.

## 7. Conclusion

In a group of patients with hypothyroidism who all were well treated by LT_4_ replacement therapy, bone turnover, density, structure, and estimated strength did not differ compared with age- and gender-matched controls in spite of differences in plasma levels of peripheral thyroid hormones nor did* in vivo* evaluation of bone at the distal radius and tibia reveal alterations in trabecular and cortical bone compartments. Our findings are reassuring for patients with well treated hypothyroidism as their disease does not seem to be associated with an increased risk of osteoporosis.

## Figures and Tables

**Table 1:** Baseline characteristics of all included patients on long-term treatment with levothyroxine and matched controls. Number of subjects (*N*) with percentages within group (%) or median with range (minimum–maximum).

	Patients (*N* = 49)	Controls (*N* = 49)	*P* value
Gender			
Male, *N* (%)	9 (18)	9 (18)	
Female, *N* (%)	40 (82)	40 (82)	
Age, yrs	55.6 (36.6–71.9)	57.3 (36.5–73.8)	0.73
BMI, kg/m^2^	27.5 (18.9–42.1)	25.7 (19.4–37.1)	0.33
Etiology			
Nontoxic goiter, *N* (%)	16 (33)		
Toxic goiter, *N* (%)	28 (57)		
Thyroid cancer, *N* (%)	5 (10)		
Duration of disease, yrs	11 (3–41)		
Treatment			
Levothyroxine, *N* (%)	49 (100)	0 (0)	
Dose, *µ*g/d	136 (50–357)	—	
Calcium, *N* (%)	21 (43)	9 (18)	**0.009**
Dose, mg/d	800 (300–1600)	800 (57–1000)	0.53
Vitamin D, *N* (%)	33 (67)	18 (37)	**0.003**
Dose, *µ*g/d	20 (5–45)	20 (1.4–50)	0.70
Magnesium, *N* (%)	5 (10)	1 (2)	**0.022**
Dose, mg/d	300 (150–370)	1400	0.33

**Table 2:** Biochemical characteristics of included patients on long-term treatment with levothyroxine and matched controls. Median with interquartile (25%–75% percentiles) range.

	Reference range	Patients (*N* = 49)	Controls (*N* = 49)	*P* value
*Plasma *				
25-Hydroxyvitamin D (nmol/L)	50–160	75.9 (55.7–93.4)	65.9 (47.6–87.5)	0.07
PTH (pmol/L)	1.6–6.9	3.7 (3.0–4.8)	4.6 (3.5–5.1)	**<0.05**
TSH (10^3^ IU/L)	0.30–4.50	2.32 (1.33–3.48)	2.00 (1.42–2.81)	0.24
T3 (nmol/L)	1.10–2.50	1.51 (1.34–1.69)	1.64 (1.47–1.86)	**<0.01**
T4 (nmol/L)	60–140	104 (94–120)	90 (78–100)	**<0.001**
T4-uptake (no unit)	0.80–1.30	1.05 (1.00–1.09)	1.04 (1.02–1.09)	0.93
T4/T3 ratio		70 (61–84)	53 (49–60)	**<0.001**
Calcium, total (mmol/L)	2.00–2.55	2.32 (2.27–2.37)	2.34 (2.28–2.40)	0.22
Calcium, ionized (mmol/L)	1.18–1.32	1.19 (1.17–1.22)	1.21 (1.18–1.23)	**<0.05**
Phosphate (mmol/L)	0.76–1.41	1.01 (0.91–1.12)	1.01 (0.86–1.14)	0.63
Magnesium (mmol/L)	0.70–1.10	0.86 (0.84–0.89)	0.84 (0.81–0.88)	**<0.05**
Albumin (g/L)	36–45	40 (38.5–42.0)	40 (38.0–41.5)	0.36
Creatinine (*µ*mol/L)	45–90	68 (61–82)	67 (61–75)	0.38
eGFR (mL/min)	>60	82 (71–89)	83 (74–90)	0.28
Bone specific alkaline phosphatase (U/L)	12–50	23.2 (19.6–29.6)	23.6 (20.5–28.6)	0.90
PINP (*µ*g/L)	20–93	43.1 (34.5–55.5)	49.4 (33.4–58.5)	0.65
Osteocalcin (*µ*g/L)	10–55	21.8 (18.4–26.8)	22.7 (17.6–28.7)	0.76
CTX (*µ*g/L)	0.03–0.84	0.39 (0.28–0.52)	0.37 (0.26–0.56)	0.87

**Table 3:** Characteristics of studied patients stratified by etiology of thyroid disease. Tests were performed by univariate ANOVA, adjusted for gender, age, and BMI. *P* values are based on *F*-tests. Mean values with 95% confidence intervals.

	Nontoxic goiter (*n* = 16)	Toxic goiter (*n* = 28)	Thyroid cancer (*n* = 5)	*P* value
*Biochemistry *				
Dose of levothyroxine, *µ*g/d^†^	107 (81–161)	136 (114–150)	171 (150–193)	0.07
TSH (10^3^ IU/L)	2.69 (2.00–3.38)	2.44 (1.92–2.96)	1.72 (0.46–2.99)	0.44
T3 (nmol/L)	1.55 (1.45–1.66)^*∗*^	1.49 (1.41–1.57)^*∗∗*^	1.61 (1.42–1.80)	0.42
T4 (nmol/L)	103 (94–113)^*∗∗*^	111 (104–118)^*∗∗*^	112 (96–129)^*∗∗*^	0.41
T4-uptake	1.06 (1.02–1.09)	1.05 (1.02–1.07)	1.04 (0.98–1.10)	0.84
T4/T3 ratio	67.5 (60.5–74.5)^*∗∗*^	75.9 (70.7–81.2)^*∗∗*^	71.4 (58.6–84.2)^*∗∗*^	0.16
Creatinine (*µ*mol/L)	73.8 (68.8–78.8)^*∗*^	68.5 (64.8–72.3)	78.0 (68.9–87.1)^*∗*^	0.08
eGFR (mL/min)	76 (71–81)	81 (77–85)	73 (64–82)	0.09
Calcium, ionized (mmol/L)	1.19 (1.17–1.21)^*∗*^	1.20 (1.19–1.22)^*∗∗*^	1.17 (1.13–1.20)^*∗*^	0.18
PTH (pmol/L)	3.69 (2.91–4.71)^*∗*^	4.27 (3.68–4.85)	3.36 (1.93–4.79)	0.32
25-Hydroxyvitamin D (nmol/L)	77.4 (66.3–88.4)^c^	69.7 (61.5–78.0)^c^	106.4 (86.3–126.5)^*∗∗*a,b^	**0.006**
*Bone structure (by HRpQCT scans), radius* ^#^				
Total area (mm^2^)	316 (287–346)^c^	299 (277–321)	250 (198–303)^a^	0.10
Total bone density (mg HA/cm^3^)	306 (270–342)	307 (280–334)^c^	378 (315–442)^b^	0.06
Cortical Area (mm^2^)	54 (47–60)^c^	56 (51–61)	67 (56–79)^a^	0.10
Cortical thickness (mm)	0.74 (0.64–0.84)^c^	0.76 (0.69–0.84)^c^	0.98 (0.80–1.16)^a,b^	0.08

^†^Kruskal-Wallis test, unadjusted.

*P* < 0.05 compared with patients with ^a^nontoxic goiter, ^b^toxic goiter, and ^c^thyroid cancer.

^*∗*^ *P* < 0.05 and ^*∗∗*^ *P* < 0.01 compared with the group of matched controls.

^#^Only indices that differed significantly between groups are shown. No differences were found at the tibia.

**Table 4:** Bone indices as measured by DXA scans in patients on long-term treatment with levothyroxine and matched controls. Median with interquartile (25%–75% percentiles) range.

	Patients (*N* = 49)	Controls (*N* = 49)	*P* value
Areal BMD by DXA, g/cm^2^			
Hip			
Total	0.902 (0.824–0.990)	0.904 (0.810–0.991)	0.90
Fem neck	0.740 (0.678–0.812)	0.724 (0.662–0.846)	0.73
Spine	1.006 (0.878–1.118)	1.029 (0.906–1.107)	0.73
Forearm			
Total	0.522 (0.478–0.583)	0.536 (0.495–0.584)	0.50
Ultra distal	0.381 (0.336–0.434)	0.387 (0.341–0.423)	0.69
MID	0.549 (0.486–0.612)	0.551 (0.518–0.602)	0.53
1/3	0.656 (0.611–0.737)	0.692 (0.629–0.732)	0.29
Whole body	1.124 (1.053–1.185)	1.151 (1.048–1.254)	0.15
Subtotal	0.942 (0.893–1.026)	0.999 (0.901–1.076)	0.11
Body composition			
Fat mass (Kg)	23.7 (17.8–29.7)	22.9 (15.7–28.0)	0.41
Lean tissue mass (Kg)	50.2 (44.7–55.9)	48.2 (44.7–54.7)	0.50

**Table 5:** Bone indices as measured by HR-pQCT scans in patients on long-term treatment with levothyroxine and matched controls. Median with interquartile (25%–75% percentiles) range.

HR-pQCT	Patients	Controls	*P* value
Distal radius (*N* = 94)			
Geometry			
Total area (mm^2^)	273.9 (248.1–344.5)	282.2 (246.5–336.0)	0.74
Cortical area (mm^2^)	57.7 (44.9–66.0)	57.7 (49.7–67.8)	0.35
Trabecular area (mm^2^)	221.0 (185.0–266.6)	221.2 (189.2–253.7)	0.87
Volumetric BMD			
Total bone density (mg HA/cm^3^)	315.1 (261.7–360.7)	330.3 (286.4–366.8)	0.56
Cortical bone density (mg HA/cm^3^)	879.2 (848.8–935.7)	899.6 (862.8–930.4)	0.50
Trabecular bone density (mg HA/cm^3^)	152.8 (125.6–179.1)	161.1 (124.4–176.4)	0.60
Meta trabecular bone density (mg HA/cm^3^)	212.0 (190.1–227.8)	219.8 (191.7–236.8)	0.48
Inner trabecular bone density (mg HA/cm^3^)	111.0 (85.6–141.0)	117.9 (83.7–136.2)	0.73
Microarchitecture			
Cortical thickness (mm)	0.78 (0.63–0.94)	0.81 (0.69–0.97)	0.41
TbTh (*µ*m)	64 (54–69)	61 (58–72)	0.89
Tb.N (mm^−1^)	2.01 (1.77–2.20)	2.00 (1.80–2.22)	0.92
Tb.Sp (mm)	0.43 (0.38–0.50)	0.43 (0.39–0.50)	0.91
TrBV/TV	0.13 (0.11–0.15)	0.13 (0.10–0.15)	0.59
Tb.N.SD (mm)	0.17 (0.16–0.21)	0.18 (0.15–0.22)	0.53
Estimated strength			
Failure load (N)	3903 (3166–4578)	3985 (3315–4625)	0.69
Stiffness (kN/mm)	82.6 (65.8–97.9)	83.4 (71.8–96.1)	0.58

Distal tibia (*N* = 98)			
Geometry			
Total area (mm^2^)	733.4 (632.2–821.7)	728.7 (661.3–814.5)	0.83
Cortical area (mm^2^)	121.8 (99.3–137.6)	123 (95.9–140.4)	0.83
Trabecular area (mm^2^)	574.6 (510.3–702.5)	614.9 (525.2–688.1)	0.76
Volumetric BMD			
Total bone density (mg HA/cm^3^)	290.9 (253.4–318.3)	279.8 (239.7–326.7)	0.98
Cortical bone density (mg HA/cm^3^)	852.7 (821.4–913.3)	860.5 (826.6–910.9)	0.94
Trabecular bone density (mg HA/cm^3^)	158.8 (135.5–182.1)	167.9 (140.2–193.2)	0.57
Meta trabecular bone density (mg HA/cm^3^)	228.0 (200.3–249.8)	232.9 (202.2–254.0)	0.61
Inner trabecular bone density (mg HA/cm^3^)	117.6 (91.5–141.8)	123.2 (96.0–150.9)	0.46
Microarchitecture			
Cortical thickness (mm)	1.17 (0.93–1.31)	1.07 (0.91–1.34)	0.72
TbTh (*µ*m)	70 (63–83)	73 (64–81)	0.92
Tb.N (mm^−1^)	1.80 (1.70–2.08)	1.88 (1.70–2.12)	0.36
Tb.Sp (mm)	0.45 (0.40–0.51)	0.48 (0.41–0.53)	0.39
TrBV/TV	0.13 (0.11–0.15)	0.14 (0.12–0.16)	0.58
Tb.N.SD (mm)	0.21 (0.17–0.24)	0.20 (0.17–0.23)	0.47
Estimated strength			
Failure load (N)	10016 (8989–11713)	10319 (8848–11867)	0.80
Stiffness (kN/mm)	210.4 (188.1–248.1)	215.6 (187.8–249.8)	0.74

Tb.N: Trabecular numbers.

Tb.Sp: Trabecular separation.

Tb.N.SD: Standard deviation of 1/Tb.N.
